# Insights into the utilisation of 1,2-propanediol and interactions with the cell envelope of *Clostridium perfringens*

**DOI:** 10.1186/s13099-025-00689-1

**Published:** 2025-04-11

**Authors:** Lucía Huertas-Díaz, Louise Guldager Vestergaard, Angeliki Marietou, Marta Irla, Jürgen Behr, Mark M. Somoza, Anders Feilberg, Clarissa Schwab

**Affiliations:** 1 Department of Biological and Chemical Engineering, Aarhus University, Aarhus, Denmark; 2 Leibniz Institute for Food Systems Biology at the Technical University of Munich, Freising, Germany; 3 Chair of Food Chemistry and Molecular Sensory Science, Technical University of Munich, Freising, Germany; 4 Department of Inorganic Chemistry, Faculty of Chemistry, University of Vienna, Vienna, Austria

**Keywords:** *C. perfringens*, 1,2-propanediol, 1-propanol *pdu*, Infant feces, Membrane composition, Membrane fluidity

## Abstract

**Background:**

Breastfeeding is a major determinant of gut microbiota composition and fermentation activity during the first months of life. Breastmilk delivers human milk oligosaccharides (HMO) as substrates for microbial intestinal fermentation. One of the main metabolites that accumulates in feces of breastfed infants is 1,2-propanediol (1,2PD) resulting from the metabolism of fucosylated HMO. 1,2PD is used in microbial cross-feeding to produce propionate, but 1,2PD is also an alcohol that can impact the state of the microbial cell envelope. To shed further light on an understudied compound in the infant gut, we investigated the genetic and metabolic potential of the early gut colonizer *Clostridium perfringens* to utilise 1,2PD, and the interactions of 1,2PD with the cell envelope.

**Results:**

Based on genome analysis, *C. perfringens* FMT 1006 isolated from infant feces possessed most genes of the *pdu* operon related to 1,2PD metabolism. *C. perfringens* consumed 1,2PD (78%) and produced 1-propanol as the main metabolite, while propionate was not detected. In agreement, genes responsible for 1,2PD utilisation and propanol formation (*pduCDE*, *dhaT*) were highly expressed. When cultivated in the presence of 1,2PD and glucose, a higher proportion of 1,2PD carbon (87%) was recovered as compared to incubation with only 1,2PD (34%). At the same time, lactate and acetate were formed in a ratio of 2.16:1.0 with 1,2PD and glucose compared to a ratio 9.0:1.0 during growth with only glucose possibly due to reallocation of the NAD^+^/NADH pool in favor of 1-propanol formation. The presence of 1,2PD slightly increased membrane fluidity and modified the composition of the membrane to a higher content of elongated glycerophosphoethanolamines.

**Conclusion:**

We provide here new knowledge on the metabolism of 1,2PD by a microbial species that is present during breastfeeding and observed that *C. perfringens* metabolised 1,2PD mainly to propanol. The presence of 1,2PD had little impact on membrane fluidity and let to modifications of membrane lipid composition. Collectively, these findings advance our understanding of on intestinal metabolite-microbe interactions during breastfeeding.

**Supplementary information:**

The online version contains supplementary material available at 10.1186/s13099-025-00689-1.

## Background

The human gastrointestinal ecosystem plays a crucial role in maintaining homeostasis and influences host health. Infants experience dynamic shifts in gut microbiota composition that are influenced by various factors including mode of birth delivery, antibiotic exposure, and diet [1]. During breastfeeding, the infant gut microbiota is exposed to various macromolecules present in human milk. Human milk oligosaccharides (HMOs) are complex and abundant carbohydrates that serve as prebiotics promoting the growth of beneficial bacteria such as *Bifidobacterium* spp. [2]. Fucosyllactose (FL) represents 90% of the HMOs in breast milk [2]. Digestion of 2-FL and 3-FL by fucosidase activity of *Bifidobacterium* spp. releases the monosaccharides glucose, galactose and fucose [3–5], which can be further metabolised to acetate, lactate, formate and 1,2-propanediol (1,2PD) through fermentation. In particular, lactate and 1,2PD accumulate in infant feces during breastfeeding in the early months of life [2] and median fecal levels of 1,2PD ranged from 5.4-14.5 mM at 3 months of age [2, 6]. 1,2PD can be metabolised to 1-propanol and the short chain fatty acid (SCFA) propionate [5]. Additionally, 1,2PD is an alcohol with the potential to inhibit microbial growth. Thus, it is crucial to understand the impact 1,2PD accumulation on the microbial community during the breastfeeding phase.

**Fig. 1:**
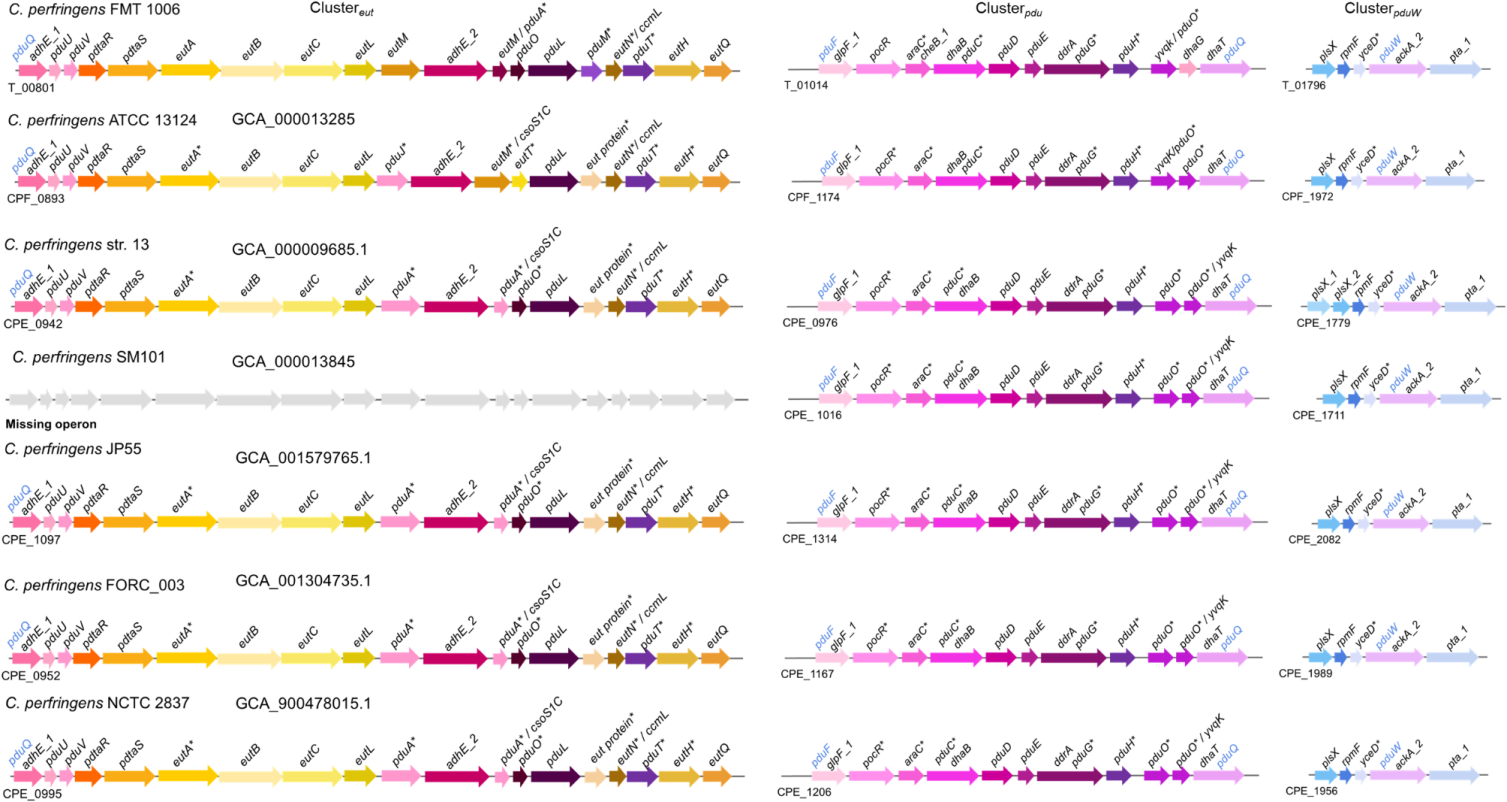
Genome arrangement of the *pdu* cluster in *Clostridium perfringens* FMT 1006 and other strains. The genome of FMT 1006 was sequenced by Oxford Nanopore Technologies and Illumina MiSeq and annotated with Prokka (annotated genes in black) with refinements using InterProScan (indicated with *). Identity of certain proteins (light blue) was additionally confirmed by BLASTp analysis. Genes were detected on three gene clusters, cluster_*eut*_, *pdu* (cluster_*pdu*_) and cluster_*pduW*_. Gray arrows indicate missing genes

Utilisation of 1,2PD is catalysed by enzymes encoded by the *pdu* operon. The signature enzyme is PduCDE, a diol/glycerol dehydratase (Fig. 1), which requires the formation of bacterial microcompartment (BMC) to protect the intracellular space from the toxic intermediate propanal [7–9]. Propanal is catabolise to propionyl-CoA by PduP and to 1-propanol by PduQ. Transformation of propionyl-CoA by PduW yields propionateand generates ATP.

1,2PD utilisation has been reported for enteropathogens such as *Listeria monocytogenes* and *Salmonella enterica* Serovar Typhimurium, and the ability to utilise 1,2PD has been linked to pathogenicity [7–9]. In the adult human gastrointestinal tract, few commensals are capable of utilising 1,2PD such as *Anaerobutyricum hallii*,* Blautia obeum*, and *Ruminococcus gnavus* [10]. During breastfeeding, these taxa only occur at low abundance in infant feces [11], at the same time, fecal propionate levels are low [12] despite the availability of 1,2PD for cross-feeding.

*Clostridium perfringens* is among the early gut colonizers with potential for PduCDE activity [2]. Strains of the species *C. perfringens* are potential toxin-carrying opportunistic pathogens, and strains can cause diseases ranging from mild gastrointestinal disturbances to life-threatening infections for example necrotizing enteritis in preterm infants, and gas gangrene [13, 14]. Yet, *C. perfringens* has been frequently detected in feces of healthy infants [2, 15, 16] and was suggested as a contributor to early butyrate production [11]. Whether the potential to use 1,2PD contributes to the occurrence of *C. perfringens* in the infant gut environment is not known.

It was the aim of this study to investigate the impact of 1,2PD on the metabolism and cell envelope of *C. perfringens* in more detail. Through a combination of genome analysis, metabolic profiling, and gene expression studies, we demonstrate that *C. perfringens* converted 1,2PD primarily to 1-propanol and that 1,2PD conversion was higher when glucose was available. The presence of the alcohol 1,2PD increased membrane fluidity and altered composition of some membrane lipids indicating that 1,2PD exerted a plethora of effects on *C. perfringens*.

## Results and discussion

### Genome characterisation and identification of *pdu* cluster

*C. perfringens* is a frequent inhabitant of the infant gut especially during the breastfeeding phase [11], yet few isolates have been characterised in detail. *C. perfringens* FMT 1006 has been isolated from infant feces [17]. To determine the genetic potential of FMT 1006, we isolated DNA and performed genome analysis combining nanopore (Oxford Nanopore Technologies) and MiSeq sequencing (Illumina). The genome was compared to other strains with closed genomes that were obtained from animals, food and the environment (Table 1). Genomes with ANI values above 95% are considered as the same species [18]. Based on our analysis using EDGAR 3.0 [19], the *C. perfringens* strains used in this study were closely related (ANI 96–99%) (Fig. S1). The pangenome encompassed 4117 genes, while the core genome consisted of 2033 genes. There were 982 singletons, and 1100 dispensable genes present in a subset of the strains.

**Table 1:** Genome characteristics of *C. perfringens* FMT 1006 (PRJEB79013) and of strains from different isolation sources. CDS: coding regions. ATCC 13,124 (GCA_000013285); Str. 13 (GCA_000009685.1); SM101 (GCA_000013845); JP55 (GCA_001579765.1); FORC_003 (GCA_001304735.1); NCTC 2837 (GCA_900478015.1). Genome assembled were obtained from NCBI, annotations were performed following the same pipeline as for FMT 1006

	*C. perfringens* strain						
	FMT 1006	ATCC 13124	str. 13	SM101	JP55	FORC_003	NCTC 2837
Accession number	PRJEB79013	GCA_000013285	GCA_000009685.1	GCA_000013845	GCA_001579765.1	GCA_001304735.1	GCA_900478015.1
Origin	Infant feces	Bovine	Soil isolate	Boiled salt beef and beans	Equine	Aquarium water	Unknown
Genome size (Mbp)	3.2	3.3	3.1	3.0	3.6	3.4	3.3
GC content (%)	28.5	28.5	28.5	28.2	28.2	28.4	28.4
Number of contigs	1	1	2	1	6	2	1
Protein-coding genes	2774	2876	2659	2578	3234	2928	2887
rRNA	30	23	30	30	30	30	30
tRNA genes	94	93	96	94	95	96	96
N50 (Mbp)	3.2	3.3	3.0	2.9	3.3	3.3	3.3
Completeness (%)	99.4	97.9	97.33	99.89	100	99.99	99.99
Contamination (%)	0.00	0.95	0.43	0.5	0.13	1.82	0.06
Coverage fold (x)	99		9.1	9.4	100	207	258
Alpha toxin	+	+	+	+	+	+	+
Reference	This article	[13]	[65]	[13]	[66]	Not published	[67]

The genome of FMT 1006 had a size of 3.2 Mbp and a GC content of 28.5% similar to the other strains (3.0-3.6 Mbp) (Table 1). FMT 1006 harboured 2774 coding DNA sequences (CDS) and a total of 124 RNA components (Table 1). FMT 1006 possessed a total of 100 unique CDS compared to the other six strains (Fig S1A). KEGG identified 1544 proteins with e-value cut-off of 0.01. All genomes possessed the *plc* gene encoding the alpha toxin Plc (phospholipase C).

Annotations of all genomes were performed with Prokka and refined with InterProScan [20, 21], which confirmed the presence of *pduCDE* genes (also sometimes annotated as *dhaB*) in *C. perfringens* FMT 1006 and the other strains encoding the three subunits of glycerol/diol dehydratases (Fig. 1). *pduCDE* (T_01016-T_01018) and *dhaT* (T_01024), encoding the 1,3-propanediol oxidoreductase (DhaT), which has been shown previously to perform alcohol dehydrogenase (PduQ) activity [22], were located in cluster with genes related to cobalamin turnover *pduGH* (T_01019-T_01020) and *pduO* (T_01021) (cluster_*pdu*_). Other potential *pdu* related genes such as *pduL (T_00814)*,* pduU* (T_00802) and *pduV* (T_00803) were found within a gene cluster that mainly contained genes related to ethanolamine utilisation (Fig. 1) (cluster_*eut*_). The gene encoding protein AdhE_1 (T_00801) that was identified as PduQ by InterProScan, was in proximity to *pduU* and *pduV* (Fig. 1). A candidate gene *pduW* (T_01799) was located at a different location on the genome (cluster_*pduW*_). When characterised with additional BLASTp analysis, PduW was 100% identical to PduW of other *C. perfringens* strains, and had 71% identity with PduW from *Clostridium baratii* (Suppl. Table 1).

We conducted additional BLASTp (v. 2.11.0+) analysis using previously functionally characterised PduP of *Salmonella* (AAD39015.1) [23] to identify possible homologues of PduP. No homologue of PduP was present in the genome of *C. perfringens*, the closest hits were AdhE_2 (T_00811) with 29% identity and another acetaldehyde dehydrogenase (T_02661) with 31% identity. Taken together, theoretical homologues of enzymes related to 1,2PD utilisation were identified with identity values above > 40% except for PduP (Suppl. Table 1).

Similar to FMT 1006, the genomes of the other assessed *C. perfringens* strains possessed most *pdu* genes except *pduP* conferring the overall potential of utilizing 1,2PD (Fig. 1). Genes were located on two clusters with the exception of *pduW* (T_01799), and gene syntety of *pdu* and *eut* was conserved except for SM101, which lacked the *eut* cluster (Fig. 1). In *Limosilactobacillus reuteri*,* Salmonella* or *Listeria*, the *pdu* genes were encoded in one operon that also included genes related to cobalamin synthesis and to ethanolamine utilisation [24–26] (Fig. 1). The *pdu* operon of *Propionibacterium freudenreichii* DSM 20271 was organised similar to *C. perfringens* with *pdu* present in two different loci [7] indicating that organisation of *pdu* differs between species.

### 1-propanol is the major compound produced from 1,2PD

As *C. perfringens* FMT 1006 possessed the general ability for 1,2PD utilisation based on its genome sequence but lacked a gene encoding PduP, which catalyses the formation of propionate, we evaluated the growth and fermentative activity in anaerobically prepared yeast extract casitone (YC) medium supplied with 50 mM glucose (YC-G50), 50 mM of 1,2PD (YC-PD50) or without additional carbon source (YC) at 37 °C for 24 h in serum flasks. The concentration of 1,2PD was chosen to reflect concentration ranges in feces [2, 6]. We measured optical density (OD_600_) and determined substrate utilisation and metabolite formation with high performance liquid chromatography with refractive index detector (HPLC-RI).

**Fig. 2:**
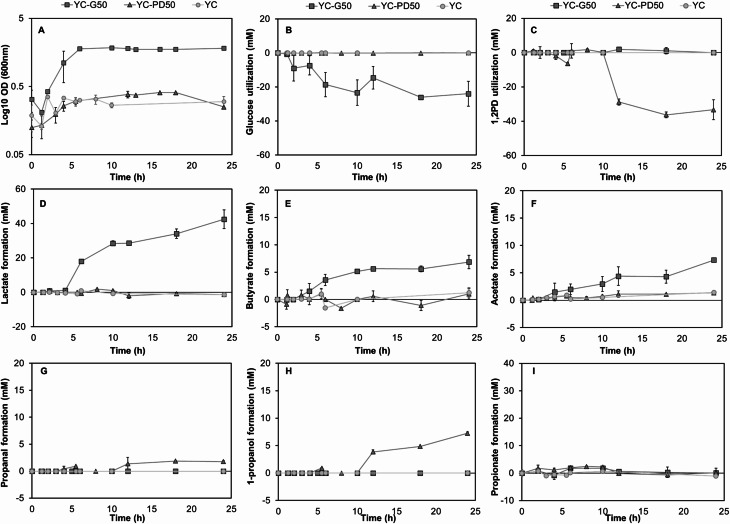
Turbidity, substrate utilisation and metabolite formation of *C. perfringens* FMT 1006. *C. perfringens* was incubated in YC-G50 (50 mM glucose) and YC-PD50 (50 mM 1,2PD) or in YC in 30 mL serum flasks for 24 h anaerobically at 37 °C, OD_600_ was monitored, and substrate utilisation and metabolite formation were analysed by HPLC-RI. (**A**) OD_600_, (**B**) glucose utilisation, (**C**) 1,2PD utilisation, and (**D**) lactate, (**E**) 1-propanol, (**F**) acetate, (**G**) propanal, (**H**) butyrate, and (**I**) propionate formation in. Each time point is an average of *n* = 2–7 biological replicates

In YC-G50, *C. perfringens* reached highest density (1.98 OD_600_) at 24 h (Fig. 2A). At 6 h, *C. perfringens* had a growth rate of 0.28 OD_600_/h and a doubling time of 2.44 h in YC-G50. *C. perfringens* reached similar OD_600_ values at 24 h when incubated in YC and in YC-PD50 (0.30 and 0.25 OD_600_, respectively) (Fig. 2A). Low optical density was also observed when *A. hallii*,* B. obeum*,* L. reuteri* or *R. gnavus* were grown in a similar medium with 1,2PD as added carbon source [10] suggesting that 1,2PD metabolism did not contribute to biomass formation.

Fermentative activity of *C. perfringens* was the highest in YC-G50. After 24 h, 42.5 ± 5.4 mM lactate was formed from 24 ± 0.9 mM glucose, followed by 6.8 ± 1.2 mM butyrate and 3.3 ± 1.6 mM acetate (Fig. 2B, D, E and F). In YC-PD50, 33.3 ± 5.8 mM 1,2PD (78.6%) were consumed and converted to 7.3 ± 0.2 mM 1-propanol and 1.8 ± 0.2 mM propanal without detectable levels of propionate (Fig. 2C, G, H and I). Propanal is a toxic compound and to prevent intracellular damage, BMC are formed during the utilisation of 1,2PD [27]. Acetate, lactate, butyrate, were present in low amounts (below 2 mM) (Fig. 2D, E and F). 1,2PD, 1-propanol, propanal or propionate were not detected when *C. perfringens* was incubated in YC (Fig. 2C, G, H and I). When *A. hallii* was tested at similar conditions, the carbon provided in form of 1,2PD was fully recovered as propanol, propanal and propionate (C_1,2PD used_=C_propanol_+C_propanal_+C_propionate_) [28], while in this study, the proportion was only approx. 34% (C_1,2PD used_ > C_propanol_+C_propanal_).

As we could not recover the expected metabolites from the liquid phase, we examined levels of 1-propanol, propanal and other potential metabolites in the headspace using proton-transfer-reaction mass spectrometry (PTR-TOF-MS) and estimated the concentrations in liquid media according to Henry’s law assuming air-water equilibrium. The detection of 1-propanol and propanal was confirmed by gas chromatography-MS (GC-MS). Smaller amounts of 2-ethyl-2-butenal and 2-methyl-pentanal were detected by both PTR-MS and GC/MS. Propanal levels were estimated 4.4-times higher based on headspace measurement than levels determined with HPLC-RI, and estimates for 1-propanol were 1.6-fold higher (Suppl. Table S2). The carbon C_1,2PD_ that could be recovered as C_propanol_+C_propanal_ was approx. 60%. As we observed a peak in HPLC-RI profiles at the retention time of 1,3-propanediol (1,3PD), we suggest that additional aldehyde hydration events occur in the liquid medium, which might account for the discrepancy between measured and estimated levels.

Taken together, our data show that *C. perfringens* metabolised 1,2PD to 1-propanol with a C_1,2PD_ recovery of 33–60% depending on analysis of compounds present in the liquid broth or the headspace.

### Gene expression profiles support observation of 1,2PD being mainly utilised to 1-propanol

Based on cultivation studies and genome characterisation, *C. perfringens* FMT 1006 did not form propionate when incubated in YC-PD50 and did not possess any genes related to propionate formation via PduP. We next determined gene expression profiles of *C. perfringens* when grown in YC-G50, YC-PD50 and YC. RNA was collected during the exponential phase of growth in YC-G50, while samples for RNA profiling of YC and YC-PD50 were obtained when the OD_600_ increased after 3 and 4 h incubation, respectively. Custom gene expression microarrays were synthesised using maskless DNA photolithography [29–31] and were hybridised using RNA transcribed to fluorescently labelled cDNA.

In YC-PD50, genes of the *pdu* cluster, i.e. *pduCDE*,* pduGH*,* pduO*,* dhaG* and *dhaT* had higher fluorescence signal (*p < 0.05*) compared with YC-G50 or YC (Table 2). At the same time, genes of the *eut* cluster_*eut*_ (*eutA*,* eutB*,* eutM* and *pduL*) had a lower signal (*p < 0.05*) in YC-PD50 compared to YC or YC-G50 (Table 2). *pduW* was not differently expressed. In YC-G50, genes related to glucose utilisation (acetyl-coA transferase *thlA*, L-lactate dehydrogenase *ldh*) or electron transfer (e.g. flavodoxin T_00556 or NADH oxidoreductase T_00907) were significantly (*p < 0.05*) upregulated compared to YC-PD50 (Table 2).

**Table 2:** Differential expression of selected genes of *C. perfringens* dependent on culture conditions. RNA isolated from *C. perfringens* FMT 1006 after 3–4 h culture in YC, YC-G50 and YC-PD50. RNA transcribed to cDNA labelled fluorescently and used in microarray hybridization. Log fold expression (LogFC) was calculated from fluorescence intensities at the tested conditions. * Indicates significancy (*p < 0.05*) with Wald’s test. n.s: not significant

genes of interest	locus_tag	YC vs. YC-PD50			YC-G50 vs. YC-PD50		
		logFC	adj. *p*. value	Change in expression level	logFC	adj. *p*. value	Change in expression level
*adhE_1*	T_00801	-0.8 to -0.1	n.s	No change	-0.5 to 0.8	n.s	No change
*pduU*	T_00802	-1.9 to 0.3	0.00*	Up in YC	-1.2 to 0.5	n.s	No change
*pduV*	T_00803	-0.8 to 0.7	n.s	No change	0.4 to 1.0	n.s	No change
*pdtaR*	T_00804	-0.5 to 0.8	n.s	No change	0.1 to 1.5	0.053	Up in YC-PD50
*pdtaS*	T_00805	-0.5 to -0.1	n.s	No change	-0.2 to 0.2	n.s	No change
*eutA*	T_00806	-2.7 to -0.4	0.00*	Up in YC	-0.7 to -1.1	0.06	Up in YC-G50
*eutB*	T_00807	-2.0 to 0.1	0.01*	Up in YC	-0.2 to 0.7	n.s	No change
*eutC*	T_00808	0.0 to 0.4	n.s	No change	0.0 to 1.1	n.s	No change
*eutL*	T_00809	-0.7 to 0.2	n.s	No change	-0.4 to 0.2	n.s	No change
*eutM*	T_00810	-1.4 to 0.2	0.03*	Up in YC	-0.1 to 0.3	n.s	No change
*adhE_2*	T_00811	0.3 to -0.4	n.s	No change	-0.4 to -0.1	n.s	No change
*eutM/pduA*	T_00812	0.2 to 0.3	n.s	No change	-0.7 to 0-0.3	n.s	No change
*pduO*	T_00813	-0.5 to 0.5	n.s	No change	-0.5 to 0.5	n.s	No change
*pduL*	T_00814	-1.5 to -0.6	0.01*	Up in YC	0.5 to -0.1	n.s	No change
*pduM*	T_00815	-0.9 to 0.2	n.s	No change	-0.5 to 0.2	n.s	No change
*eutN/ccmL*	T_00816	-1.2	n.s	No change	-0.7	n.s	No change
*pduT*	T_00817	-0.7 to -0.1	n.s	No change	-0.6 to 0.5	n.s	No change
*eutH_2*	T_00818	-0.9 to -0.1	n.s	No change	-1.2 to -0.2	n.s	No change
*eutQ*	T_00819	-0.8 to -0.2	n.s	No change	-0.9 to -0.3	n.s	No change
*glpF_1*	T_01014	0.5 to 1.4	0.03*	Up in YC-PD50	-0.1 to 0.2	n.s	No change
*pcoR*	T_01015	-0.8 to 1.2	n.s	No change	-1.6 to 0.6	n.s	No change
*araC*	T_01016	-0.1 to 0.6	n.s	No change	-0.5 to -0.3	n.s	No change
*pduC*	T_01017	0.3 to 1.4	0.057	Up in YC-PD50	1.1 to 0.1	n.s	No change
*pduD*	T_01018	1.6 to 2.9	0.01*	Up in YC-PD50	1.3 to 0.5	n.s	No change
*pduE*	T_01019	1.1 to 2.3	0.00*	Up in YC-PD50	0.5 to 1.0	n.s	No change
*pduG*	T_01020	0.4 to 2.9	0.01*	Up in YC-PD50	1.0 to 1.2	0.053	Up in YC-PD50
*pduH*	T_01021	-0.5 to 1.6	0.046*	Up in YC-PD50	-0.7 to 0.2	n.s	No change
*pduO*	T_01022	1.4 to 3.4	0.02*	Up in YC-PD50	0.7 to 1.7	0.085	Up in YC-PD50
*dhaG*	T_01023	1.8 to 1.9	0.00*	Up in YC-PD50	0.4 to 1.3	0.058	Up in YC-PD50
*dhaT*	T_01024	1.6 to 2.4	0.00*	Up in YC-PD50	1.8 to 0.5	0.05	Up in YC-PD50
*plsX*	T_01796	-0.9 to 0.0	n.s	No change	-0.8 to -0.5	n.s	No change
*rpmF*	T_01797	-0.2 to -0.1	n.s	No change	0.1 to 0.5	n.s	No change
*yceD*	T_01798	-0.3 to 0.6	n.s	No change	-0.3 to 0.5	n.s	No change
*ackA_2 (pduW)*	T_01799	-1.3 to -0.1	n.s	No change	-1.2 to 0.4	n.s	No change
*pta*	T_01800	-1.7 to -0.1	n.s	No change	-2.1 to -0.4	n.s	No change
*ldh*	T_00063	-0.5 to 0.4	n.s	No change	-1.4	0.00*	Up in YC-G50
flavodoxin	T_00556	-1.6 to 0.6	n.s	No change	-3.3 to -3	0.00*	Up in YC-G50
NADH(P) oxidoreductase	T_00907	-0.6	n.s	No change	-1.4	0.03*	Up in YC-G50
*thlA*	T_02278	-0.2 to 0.8	n.s	No change	-3.1 to -2.5	0.00*	Up in YC-G50

We conducted additional quantitative PCR (qPCR) analysis to determine expression levels of *pduC*,* pduD*,* dhaT* and *pduL* relative to housekeeping genes *ftsZ*, *recA* and *gyrA*. In agreement with microarray analysis, *pduC*,* pduD* and *dhaT* were 0.06–0.09 fold downregulated in YC relative to YC-G50, while the same genes were upregulated 2.7–5.6 fold in YC-PD50 relative to YC-G50 (Table 3). *pduL* was 2.6-fold higher expressed in YC relatively to YC-G50 (Table 3).

**Table 3:** Relative expression of selected *pdu* genes. RNA isolated from *C. perfringens* FMT 1006 after 3–4 h cultivation in YC, YC-G50 and YC-PD50. cDNA was prepared from RNA and gene expression was determined by qPCR using cDNA as template. For standardisation, expression of target genes was calculated relative to the geometric mean of housekeeping genes *ftsZ*,* gyrA* and *recA*, and to expression in YC-G50. RNA was collected from two independent experiments, and each sample was analysed in technical duplicates

Targeted genes expression relative to housekeeping genes relative to expression in YC-G				
Medium / gene	*pduC*	*pduD*	*dhaT*	*pduL*
YC	0.09 ± 0.02	0.06 ± 0.03	0.07 ± 0.01	2.61 ± 0.75
YC-G50	1.00 ± 0.44	1.04 ± 0.54	1.02 ± 0.70	1.02 ± 0.42
YC-PD50	2.75 ± 0.02	5.32 ± 0.02	2.72 ± 0.26	1.25 ± 0.26

### The impact of glucose on 1,2PD metabolism

As we observed that the addition of 1,2PD did not support growth of FMT 1006 and that the intermediate propanal was detected after 24 h of incubation, we determined if glucose addition improved biomass formation and the metabolism of 1,2PD when supplied at different concentrations. We combined glucose and 1,2PD at equimolar concentrations (YC-PD10-G10 and YC-PD50-G50) and compared to YC-PD50. FMT 1006 was cultured for 24 h at 37 °C, and turbidity was determined using a McFarland densitometer. HPLC-RI was used to determine substrate utilisation and metabolite formation.

**Fig. 3:**
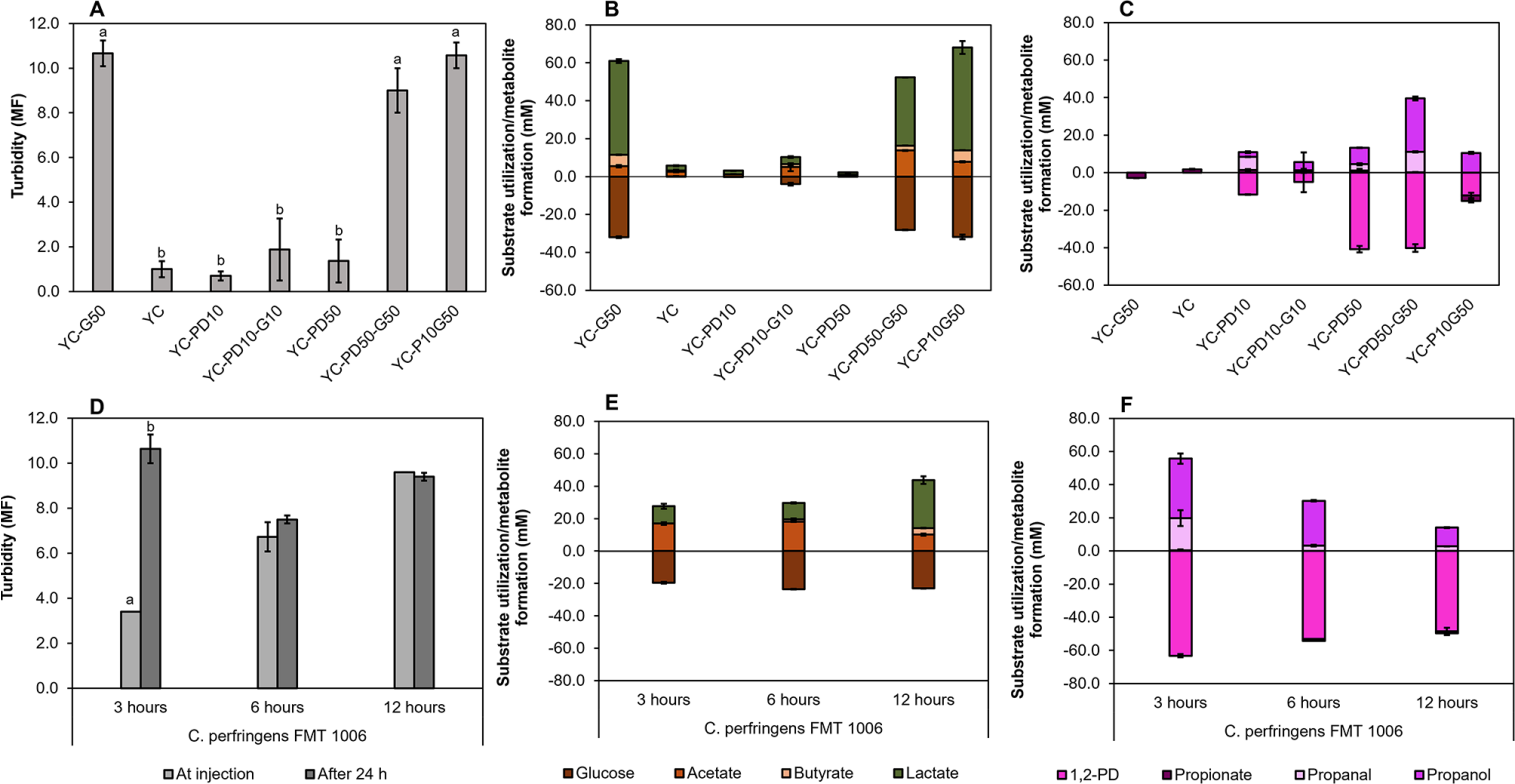
Impact of glucose on 1, 2PD metabolism of *C. perfringens* FMT 1006. *C. perfringens* was cultured in YC-G50, YC, YC-PD10, YC-PD10-G10, YC-PD50, YC-PD50-G50) (**A, B,C**) or in YC-G50 with addition of 1,2PD at 3, 6, and 12 h (**D, E, F**) for 24 h at 37 °C. Density was monitored using McFarland densitometer (MF) and substrate utilisation and metabolite formation was recorded with HPLC-RI. (**A, D**) Turbidity measurements in McFarland units (MF). Significant difference between turbidity values was determined by Dunn’s Test adjusted by Holm. A *p < 0.05* was considered significant and was indicated with different letters, (**B, E**) Utilisation of glucose and formation of acetate, lactate and butyrate. (**C, F**) 1,2PD utilisation and formation of 1-propanol and propanal. Shown are average values from from biological triplicates and standard deviations

Final turbidity was low in YC-PD10-G10 (1.9 ± 1.4 McFarland units, MF), YC (1 ± 0.4 MF), YC-PD10 (0.7 ± 0.2 MF) and YC-PD50 (1.4 ± 1 MF). When glucose was supplemented to YC-PD50 (YC-PD50-G50, 9 ± 1 MF), turbidity was similar to YC-G50 (10.7 ± 0.6 MF) (Fig. 3A). Compared to cultivation in YC-G50, the presence of 50 mM 1,2PD (YC-PD50-G50) shifted the ratio of acetate:lactate from 1.0:9.0 to 1.0:2.6 (Fig. 3B). Low concentrations of 1,2PD (YC-PD10) led to higher yields of propanal from 1,2PD (60.2% mM_propanal_/mM_1,2PD_) compared to YC-PD50 (8.3% mM_propanal_/mM_1,2PD_). In contrast, the proportion of 1-propanol was similar in YC-PD10 and YC-PD50 (21.0% mM_propanol_/mM_1,2PD_) (Fig. 3C). When glucose was present, the proportion of propanal and 1-propanol was 12% and 88% and 27.1 and 70.1% in YC-PD10-G10 and YC-PD50-G50, respectively (Fig. 3C).

**Fig. 4:**
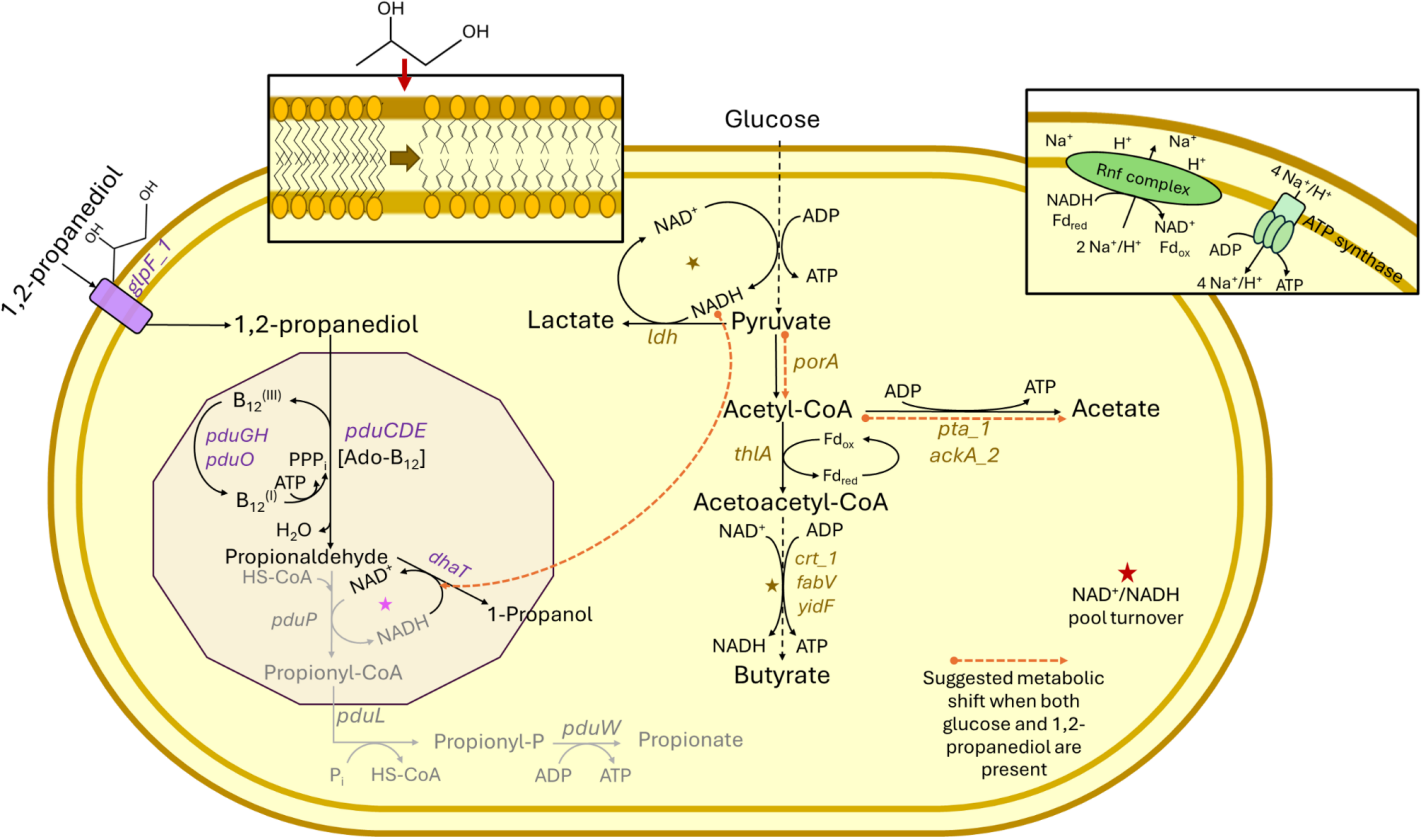
Genome based pathway reconstruction supported by gene expression analysis and metabolite data. Suggested schemes of metabolic pathways in the presence of 1,2PD and glucose in *C. perfringens* FMT 1006. Stars indicate proposed NAD^+^/NADH pools for metabolisms examined during this study. Orange dashed arrow indicates the proposed metabolism shift when 1,2PD and glucose are supplied simultaneously in *C. perfringens* FMT 1006

Taken together, we observed that propanal production did not impact final turbidity (*p > 0.05*) when glucose was provided together with 1,2PD even at 50 mM, suggesting that the low biomass observed in the absence of glucose may not be due to propanal production. Within the Pdu pathway, conversion of propanal by PduQ and PduP requires NAD^+^/NADH as co-factors (Fig. 4). PduQ uses NADH as electron donor, while PduP employs NAD^+^ as electron acceptor [32, 33]. In previous studies, *Salmonella* formed 1-propanol and propionate in a ratio of 1:1 [34, 35]. In contrast, *C. perfringens* converted 1,2-PD to 1-propanol without concomitant production of propionate. In our genome-based pathway model, *C. perfringens* only possessed DhaT producing NAD^+^ and lacked NADH-generating PduP (Fig. 4). Lactate production was highest during growth in YC-G50, and lower lactate production in the presence of both glucose and 1,2PD may be associated with DhaT activity, which used NADH as electron donor instead of lactate dehydrogenase Ldh (Fig. 4). Similarly *A. hallii* produced more 1-propanol (and not propionate) when grown in the presence of 1,2PD and glucose, compared to 1,2PD alone [28] suggesting that *A. hallii* also regenerated NAD^+^ pool through activity of PduQ.

To investigate further whether 1-propanol formation depended on the concurrent formation of acetate and/or lactate, we evaluated the impact of addition of 1,2PD (50 mM) to cultures grown in YC-G50 at early exponential (3 h), late exponential (6 h) and during stationary growth phase (12 h). After 24 h growth, highest turbidity was achieved when 1,2PD was added at 3 h (10.6 ± 0.6 MF), followed by 12 h (9.4 ± 0.2 MF) and 6 h (7.5 ± 0.2 MF) (Fig. 3D). Turbidity recorded at 3 h before injection (3.4 ± 0.0 MF) was lower (*p < 0.05*) compared to 24 h (Fig. 3D). Consumption of glucose (19.6–23.6 mM) and 1,2PD (48.4–63.2 mM) was similar at all three conditions, suggesting that substrate utilisation was not affected by the concurrent presence of glucose and 1,2PD (Fig. 3E and F). These observations are in contrast to *Clostridium beijerinckii* or *C. butylicum*, whose Pdu metabolism was repressed when glucose was provided [36, 37].

When 1,2PD was added after 3 or 6 h, levels of acetate and lactate were similar, and the ratio of acetate: lactate was 0.6:1.0 compared to 2.9:1.0 at 12 h (Fig. 3E). When 1,2PD was added at 3 h, the recovery of C_1,2PD_ used as C_propanol_+C_propanal_ was 87% with 35.9 ± 3.0 mM 1-propanol and 19.3 ± 4.7 mM propanal produced (Fig. 3F). At 6 and 12 h, the recovery of C_1,2PD used_ as C_propanol_+C_propanal_ was lower (57% and 29%, respectively) (Fig. 3F). Conversion to 1-propanol was higher (*p < 0.05*) when 1,2PD was added during exponential growth compared to stationary phase.

These differences in recovery of 1,2PD metabolites again suggest that the conversion of pyruvate to acetate or lactate plays a role in the production of 1-propanol. In the presence of glucose, acetate formation can deliver ATP (Fig. 4), and when pyruvate is transformed to acetate, there is less lactate formed, and NADH consumed which can provide NADH to PduQ (Fig. 4). In infant feces, *C. perfringens* likely has access to both glucose and 1,2PD. In agreement, 1-propanol was recovered in 54-63% of fecal samples of breastfed infants that were collected between 2 weeks and 2 months of life [38, 39]. 1-propanol was detected in feces of breastfed infants as early as eight days after birth [39], in agreement with early colonisation by *Clostridium* spp. [40]. While *C. perfringens* might not be able to contribute to the intestinal propionate pool through 1,2PD cross-feeding, it can provide 1-propanol to the gut intestinal environment.

### 1,2PD had little impact on membrane fluidity of *C. perfringens*

At the provided cultivation conditions, *C. perfringens* FMT 1006 growth was not supported by the presence of 1,2PD. As an alcohol, 1,2PD could increase in membrane fluidity, leading to leakage of cofactors and even a collapse of metabolic activity [41]. Ethanol and butanol have been shown to decrease proton flux across the membrane, to alter ATPase activity and to reduce energy production [41]. Therefore, we investigated the effect of 1,2PD on the state of the membrane using the stain Laurdan (1-(6-(dimethylamino)naphthalen-2-yl)dodecan-1-one). Due to its apolarity, Laurdan can dissolve within the lipidic membrane, shifting emission spectra from 440 nm (gel-phase) to 490 nm (liquid-phase). We used phenylethanol (PE) (10 and 50 mM) as positive control as PE increases fluidity [42], and tested 1,2PD (10 and 50 mM) and the pathway intermediate propanal (20 and 50 mM).

**Fig. 5:**
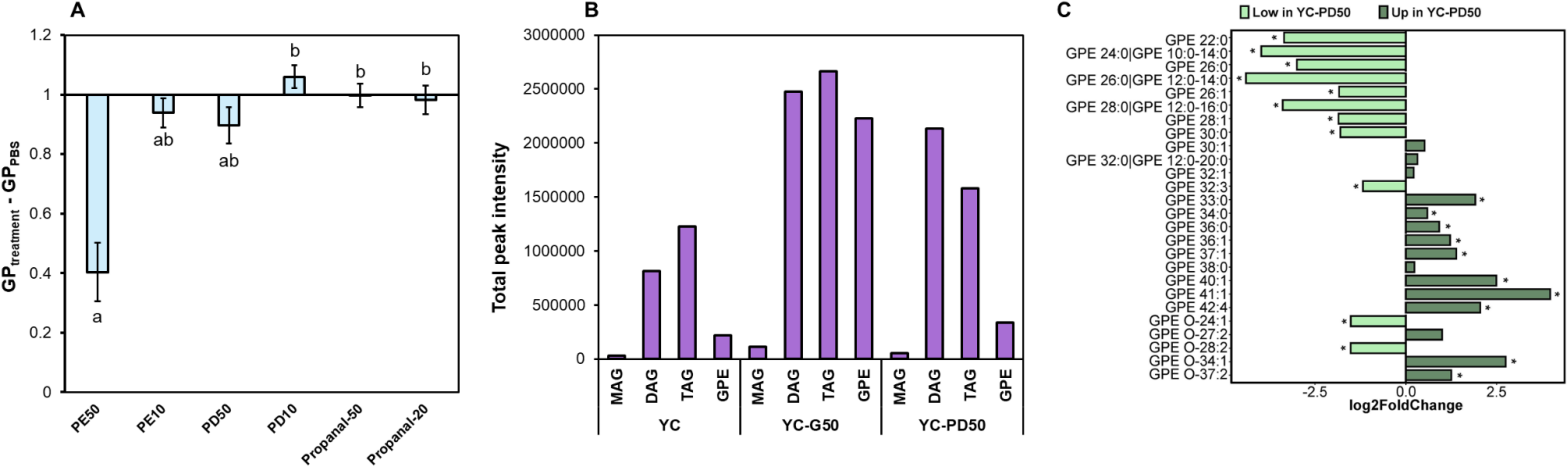
Impact of 1,2PD on the membrane fluidity and composition of *C. perfringens*. The impact of 1,2PD on membrane fluidity was determined using Laurdan while composition of membrane lipids after incubation in YC, YC-G50 and YC-PD50 for 24 h at 37 °C was analysed using LC-MS/MS. (**A**) Effect of 1,2PD on general polarisation (GP) of the membrane. Shown are the differences in GP levels in treatment versus the control (PBS only). Experiments were conducted in biological triplicates. (**B**) Peak intensities of major membrane lipid components. Shown are mean values of biological duplicates. (**C**) Log2 fold change of lipids in YC-PD50 (50 mM) compared to YC-G50 (50 mM) obtained from DESeq analysis in *C. perfringens* FMT 1006 cultured in YC-G50 (50 mM) or YC-PD50 (50 mM). Different letters denote significant difference between conditions (*p < 0.05*, Dunn’s Test adjusted by Holm). Significance by Wald test (*p < 0.05*) is indicated with (*). PE: Phenylethanol. PD: 1,2-propanediol. Diacylglycerols (DAG), monoacylglycerols (MAG), glycerophosphoethanolamines (GPE) and triacyclglycerols (TAG)

Addition of PE significantly increased (*p < 0.05*) membrane fluidity in a dose-dependent manner compared to cells that were not treated as indicated by a lower Generalised Polarisation (GP) value. In comparison, 1,2PD increased membrane fluidity less than 0.9-fold when added at 50 mM, and reduced membrane fluidity by ∆GP 1.05 at 10 mM (Fig. 5A). The aldehyde propanal did not affect overall membrane fluidity. Huffer et al. [41] assessed the effect of different alcohols on bacterial membranes, and concluded that strains whose membrane fluidity did not change significantly were more tolerant towards alcohol exposure. These tolerant strains included *C. beijerinckii* [41]. As there was only a slight effect on membrane fluidity even at a concentration of 50 mM, our results point out that the *C. perfringens* FMT 1006 membrane was tolerant towards exposure to 1,2PD at levels that might be similar as observed in vivo [2, 6].

### Incubation in the presence of 1,2PD altered composition of a fraction of membrane lipids

Exposure to alcohols might not only affect membrane fluidity but can also cause alterations in lipid composition. We therefore evaluated membrane composition of *C. perfringens* FMT 1006 after 24 h incubation in YC-PD50, YC and YC-G50. We extracted the lipidic fraction using the SIMPLEX protocol [43] and determined lipid composition with liquid chromatography–tandem mass spectrometry (LC-MS/MS). For data extraction, peak identification and normalisation we used MS-DIAL [44] and further analysed the results with RStudio.

Membrane lipids were mainly composed of diacylglycerols (DAG), monoacylglycerols (MAG), triacyclglycerols (TAG), and glycerophosphoethanolamines (GPE) (Fig. 5B). During growth in YC-G50, the most abundant lipids were DAG (33%), TAG (36%) and GPE (30%). When cultured in YC and YC-PD50, the proportion of GPE (10% and 8%) was lower compared to YC-G50, while the proportions of TAG (54% and 38%) and DAG (36% and 52%) were higher (Fig. 5B). There was little difference in the composition of the MAG and DAG fractions when *C. perfringens* was incubated in YC-G50 or YC-PD50 (data not shown). In contrast, the composition of GPE lipids, which are major components of the lipidic membranes of gram positive bacteria [45], differed (Fig. 5C).

There were little difference in the composition of the MAG and DAG fractions when incubated in YC-G50 and YC-PD50 (data not shown) while the composition of GPE lipids shifted (Fig. 5C). The membrane of *C. perfringens* FMT 1006 had a higher abundance of shorter GPE when cultured in YC-G50 compared to YC-PD50 (Fig. 5C) suggesting a more pronounced membrane rigidification due to the presence of 1,2PD (Fig. 5C) [45].

As we observed changes in membrane lipid composition, we investigated expression of genes of the fatty acid biosynthesis cluster *fab*, which has been linked to membrane lipid modification during stress conditions in *Bacillus* and *Clostridium* sp [46, 47]. Based on microarray data, expression levels of fab related genes did not significantly differ between growth in YC-PD50 compared to YC or YC-G50 (Suppl. Table S3).

## Conclusion

Composition of the intestinal microbiota of infants and its fermentation activity is increasingly recognised as contributor to health outcome in later life, yet the role of the alcohol 1,2PD during breastfeeding has been little studied. Our results suggest that *C. perfringens* is a utiliser of 1,2PD that can contribute to intestinal 1-propanol formation during breastfeeding. The presence of 1,2PD altered composition of certain membrane lipids and caused little changes of membrane fluidity, which might indicate a certain level of tolerance of *C. perfringens* towards 1,2PD. These findings advance our understanding of the interactions of intestinal metabolites and microbes during breastfeeding. Currently, few strains of *C. perfringens* have been isolated from infant feces and characterized in regard to 1,2PD utilisation; investigations on further strains will indicate the potential to generalise findings made for FMT 1006.

## Materials and methods

### Culture media and incubation conditions

Materials were purchased from Merck unless stated otherwise. *C. perfringens* FMT 1006 was obtained from the strain collection of the group of Functional Microbe Technology (FMT) (Aarhus University, Denmark) and was originally isolated from infant feces [17]. FMT 1006 was routinely cultivated in Wilkins-Chalgren medium supplemented with 5 g/L soya peptone (WCSP, Thermo Fisher), 1 g/L Tween 80, and 0.5 g/L L-cysteine-HCl. The pH was adjusted to 7.1 before boiling, and L-cysteine-HCl was added before degassing for a final pH of 6.5 after autoclaving. Samples were dispersed in serum flasks (30 mL) or Hungate tubes (10 mL) under constant CO_2_ flow, closed and autoclaved at 120 °C for 20 min. For initiation of working cultures, cells were streaked from frozen stocks on anaerobically prepared WCSP agar plates (WSCP with the addition of 15 g/L agar (VWR)) in an anaerobic chamber (10% CO_2_, 5% H_2_, 85% N_2_, Baker Ruskinn) and were incubated at 37 °C for 24 h.

Colonies were transferred to a 10 mL Hungate tube containing anaerobically prepared, modified yeast extract-casitone (YC) medium as described by Huertas-Díaz et al. [2]. supplied with 50 mM glucose (YC-G50). After two transfers in YC-G50, cultures were used in the assays. Strain purity was routinely checked using bright field microscopy.

To determine growth behaviour and to collect samples for RNA isolation and analysis of membrane lipids, working cultures were inoculated (2%) in YC in serum flasks in YC, YC-G50 and, YC-PD50. Growth was monitored as optical density at 600 nm (OD_600_) using a cell densitometer (Ultrospec 10, Biochrom). When incubated with glucose, *C. perfringens* showed regular growth behaviour with a lag phase, exponential and stationary growth phase, while during incubation in YC and YC-PD50, there was only a minor increase in turbidity at around 3–4 h. We therefore collected cell pellets of cultures grown in YC-G50 at early-stage growth phase (0.2 OD_600_), for isolation of RNA. Samples for RNA profiling of YC and YC-PD50 were obtained when the OD_600_ increased from 0.14 to 0.20 and from 0.16 to 0.30 after 3 and 4 h incubation, respectively. Cell pellets were snap frozen in liquid nitrogen and placed at − 80 °C until further processing. All experimental work was performed in independent biological replicates (*n* = 2–7). Cell pellets were collected from the remaining biomass after 24 h incubation for determination of the composition of membrane lipids and stored at − 80 °C.

To test for the impact of glucose on 1,2PD metabolism, working cultures were inoculated (2%) in Hungate tubes containing YC-G50, YC, YC-PD10, YC-PD10-G10, YC-PD50, YC-PD50-G50. Additionally, we investigated how 1,2PD addition affected the fermentation activity of *C. perfringens* grown in YC-G50 and added 1,2PD after 3, 6 and 12 h of incubation. Turbidity was measured prior addition of 1,2PD and after 24 h incubation. Growth rate in YC-G50 was calculated at 6 h of growth as 1/(duration of incubation * ln [2]) / ln (OD_600_/initial OD_600_).

### DNA isolation and genome analysis

DNA was extracted from working cultures following the instructions from manufacturer (Genomic DNA purification kit GeneJET, Thermo Scientific for Gram-positive bacteria). Purified DNA was suspended in 40 µL of TE buffer. Libraries were prepared and sequenced using MiSeq (Department of Biology, Aarhus University) and Oxford Nanopore Technologies (Plasmidosaurus) and standard library preparation protocols. For quality filtering and assembly, the following process was used: FitLong (v0.2.1) was used to remove 5% of the lowest quality reads and to reduce the reads to 250 Mb [48]. A rough assembly was created with Miniasm (v0.3) which reduced to 100× coverage [49]. Flye (v2.9.1) assembly was run with parameters selected for high quality ONT reads [50] and Medaka (v1.8.0) was used to refine the Flye assembly [51]. Contig analysis was performed using Bandage (v0.8.1), quality check and contamination was assessed by CheckM (v1.2.2) [52, 53]. The assembly was refined using Polypolish (v0.5.0) with the Illumina reads previously trimmed with Trimmomatic (v0.39) and quality checked with FastQC (v0.11.9) [54–56]. Annotations were done with Prokka (v1.14.5) [20] and InterProScan (v5.60-92.0) was used for annotation refinements [21]. Metabolic pathways were assessed with KofamKOALA (v2024.02.01) [57]. Annotations were evaluated using Artemis software (v18.2.0) [58]. Additional BLASTp was performed with selected Pdu proteins of FMT 1006 against the NCBI using the database (non-redundant protein sequences). For comparative genome analysis of *C. perfringens* FMT 1006 and the complete genomes of six other strains of *C. perfringens* (Table 1), EDGAR3.0 was used [19] to determine the core genome ANI values based on all-against-all comparisons [18, 19].

### RNA extraction and cDNA labelling

To isolated RNA, we followed TRizol procedure. TRizol (1 mL) was added to the pellets and incubated at room temperature for 5 min. Chloroform (0.2 mL) was added, mixed by shaking, and the sample was incubated for 3 min, followed by centrifugation for 15 min at 12 000 × g at 4 °C. The aqueous phase was transferred into a new 1.5 mL tube and mixed with an equal volume of 70% ethanol. The sample was transferred to a RNeasy spin column and processed according to the manufacturer’s instruction (RNeasy Mini Kit, Qiagen). Chromosomal DNA was removed using the TURBO DNA-free™ Kit (Thermo Fisher) and the absence of DNA was confirmed using quantitative PCR (qPCR) with primers targeting the 16 S rRNA gene as described [59]. A total of 10 µg of RNA were simultaneously reverse transcribed and labelled using 7 µg of 5′ Cy3 random nonamer (Biomers) as previously described [30]. Briefly, the mixture was diluted in nuclease free water and incubated for 70 °C for 5 min and placed immediately on ice. For reverse transcription, a mixture of 6 µL of 5X first strand buffer, 1.5 µL of 0.1 M DTT, 1 µL of 10 mM dNTP, 4 µL of Superscript 3 (200 U/µL, Thermo Fisher) and 1 µL of RNase out (40 U/µL, Thermo Fisher) was added to the previous mixture and incubated for 5 mi at 25 °C followed by 3 h at 42 °C. For hydrolysis, a mixture of 16 µL of 200 mM NaOH and 16 µL of 20 mM of EDTA were added to the mixture and incubated for 10 min at 65 °C. For the neutralisation step, 64 µL of 1 M HEPES (pH 7) was added to the previous mixture. For purification, QiaQuick PCR purification kit was used according to instructions.

### Microarray hybridisation and quantitative PCR

Probes for the *C. perfringens* were designed using OligoPicker software [60] with default settings. For microarray design a total of 2618 unique oligo sets out of total 2771 genes. Each gene was represented for an average of 2.5 different probes of 70mer length. A 4-part chamber array design was used with 35.000 spots from which each probe would be represented approximately 5 times and each gene 12.5 times. 60 probes were added with ‘QC 25mer’ as an internal quality control and 10 empty sequences for background subtraction. For gene expression analysis, 0.125 µg of Cy3-labelled cDNA were used per chamber in a 4-part array. Data extraction was performed using NimbleScan 2.1 (Roche-NimbleGen), employing robust multichip analysis for inter-array normalization purposes. All experiments were performed in biological and technical duplicates. Data normalisation was performed using robust multichip analysis (RMA) method followed by quality control of QC25 k-mers [61]. Background probes were added to all parts of microarray and background was subtracted after normalisation. For final data comparison, DESeq2 analysis was used [62]. Further information is provided in supplementary methods. qPCR was performed to determine relative expression levels of major genes of *pdu* as described in the supplementary methods [59].

### Membrane fluidity assay

To investigate changes in the fluidity of the cell membrane, a Laurdan staining method was adapted taking into account the requirements for anaerobic conditions of FMT 1006. All procedures were performed in an anaerobic chamber and all chemicals and materials were pre-heated to 37 °C to avoid membrane-associated thermal changes. Laurdan stock (1 mM) in acetone was kept at -20 °C.

Samples (0.5 mL) were withdrawn from a 24 h culture in YC-G50 and mixed with PBS buffer (pH 7.4) to a final density of 0.5 OD_600_. The cell pellet was washed with phosphate buffered saline (PBS) twice at 10,000 × g for 30 s. Samples were resuspended in PBS containing 50 mM 1,2PD (PD50), 10 mM 1,2PD (PD10), 50 mM PE, 50PE, 10 mM PE (10PE), 50 mM propanal, 20 mM propanal, or PBS only as negative control together with Laurdan (10 µM). Samples were incubated for 30 min at 37 °C.

After incubation, cells were washed twice with PBS buffer and pellets were resuspended in the same treatment solutions without Laurdan. Cell suspensions (200 µL) were added to a 96-well black clear bottom plate and fluorescent intensity was determined using a fluorescence microplate reader (CLARIOstart Plus, BMG LABTECH) at excitation 350 nm and emission 380 nm to 600 nm at 37 °C. The experiment was repeated three times, and each sample was analysed in technical duplicates.

The Generalised Polarisation (GP) values were calculated using fluorescent emission intensity values at 440 nm and 490 nm as follows: GP=(I_440_-I_490_)/(I_440_ + I_490_).

### Determination of changes in membrane composition

To extract membrane lipids, we followed the SIMPLEX method for lipidomics as described [43] followed by LC-MS/MS for identification. Eluent A was prepared with MeOH/ACN/H2O (1:1:3), 5 mM ammonium acetate, 10 nM EDTA. Eluent B contained isopropanol with ammonium acetate (5 mM) and EDTA (10 nM). A 2 µL injection under a flow rate of 200 µL/min. Using a column Kinetex 1.7 μm C18 100 Å size 100 × 2.1 mm. Instrument control and data processing software was performed by Analyst (TF 1.7.1). Data processing was performed using MS Dial for lipidomics. Only proton adduct was analysed by MSDial [44]. Data was extracted after normalisation and analyzed by DESeq2 package in R. Analysis performed from biological duplicates. Further information in Supplementary materials and methods.

### Substrate, metabolite and gas analysis

Substrate levels and fermentation metabolites present in supernatants were analysed by high performance liquid chromatography coupled to a refractive index detector (HPLC-RI) using a 1260 Infinity II LC System (all Agilent). Compounds were separated using a Hi-Plex H column (300 × 7.7 mm) attached to a guard (50 × 7.7 mm) column. The samples (10 µL injection volume) were eluted with 5 mM H_2_SO_4_ at a flow rate of 0.6 mL/min at 40 °C. Fermentation metabolites were quantified using external standards.

PTR-TOF-MS measurements were performed as previously reported [63]. The system was operated with H_3_O^+^ as the primary ion using the proton affinity to detect compounds by direct time-of-flight mass spectrometry [63]. Settings were 2.4 mbar, voltage 600 V and a temperature 80 C for an E/N of 140 Td The can mode ranged from m/z 5 to 200 and the airflow was set to 400 mL/min After calculating of concentrations within the headspace, Henry’s law was applied to calculate equilibrium liquid concentrations [63]. In addition, headspace gas phase sampling was done on adsorbent tubes coupled to thermal-desorption gas chromatography with mass spectrometric detection (TDGCMS) to verify identification of unknowns. The method has been described in more detail before [64].

### Statistics

DESeq2 analysis was used both for microarray analysis and for lipidomics comparison [62]. Significance was calculated by Wald test. For statistical analysis of turbidity, we employed Kruskal-Wallis test with posthoc Dunn’s test adjusted by Holm using the package ‘rstatix’ v.0.7.2. Figures were made with RStudio and posterior modifications by Adobe Acrobat Pro, Excel or PowerPoint.

## Electronic supplementary material

Below is the link to the electronic supplementary material.

Supplementary Material 1

## Data Availability

The genome of *C. perfringens* FMT 1006 is assessable at the ENA under accession number PRJEB79013. Microarray data is available at Zenodo at accession number 14244879.
