# Supplementary material for: Insights into the utilisation of 1,2-propanediol and interactions with the cell envelope of Clostridium perfringens
Source: Gut Pathog. 2025 Apr 11;17:23. doi: 10.1186/s13099-025-00689-1 (PMC11992839; doi:10.1186/s13099-025-00689-1)
Supplement: Supplementary file 1 — Supplementary Material 1 [file 13099_2025_689_MOESM1_ESM.docx]

# Supplementary data for

Insights into utilisation of 1,2-propanediol and interactions with the cell envelope of *Clostridium perfringens*

**Running title**: Interactions of 1,2-propanediol with *Clostridium perfringens*.

Lucía Huertas-Díaz^a^, Louise Guldager Vestergaard^a^, Angeliki Marietou^a^, Marta Irla^a^, Jürgen Behr^b^, Mark M. Somoza^b,c,d^, Anders Feilberg^a^, Clarissa Schwab^a^

^a^Department of Biological and Chemical Engineering, Aarhus University, Denmark

^b^Leibniz Institute for Food Systems Biology at the Technical University of Munich, Freising, Germany

^c^Chair of Food Chemistry and Molecular Sensory Science, Technical University of Munich, Freising, Germany

^d^Department of Inorganic Chemistry, Faculty of Chemistry, University of Vienna, Vienna, Austria

### Supplementary methods

### Silanization of glass slides

Glass microscope slides (Schott Nexterion D 263) were used according to Das, Santhosh *et al*. 2023 (1). Briefly, slides were functionalized using EtOH/H_2_O (95:5, v/v) containing 0.2% acetic acid with 32.5 mmol of dipodal monohydroxyl silane. Then, they were placed into a stainless-steel-rack and incubated into a solution of dipodal monohydroxyl silane for 4 hours at RT under gentle agitation. Afterwards, the slides were washed twice with EtOH/H_2_O (95:5, v/v) and 0.2% acetic acid for 20 minutes, drained wash solution and dried under argon to remove residual droplets. Followed by 2 hours dried in a vacuum oven at 120°C and left overnight to cool down under vacuum. Slides were stored in the desiccator until further use. For generating the DNA microarrays, in situ chemical photolithography was used by using a maskless array synthesiser, consisting of an optical system synchronized with a chemical delivery system, as previously described (1).

### Microarray hybridization and quantitative PCR

Custom gene expression microarrays were synthesized using maskless DNA photolithography (1–3). The microarray design included an average of 2.5 70-mer probes per gene for 2618 out of total 2771 genes long enough to yield valid probes. Each microarray included 35 000 spots consisting of an average of 12.5 replicates per gene as well as microarray synthesis quality control probes and empty probes as a background control. The microarrays were hybridized using RNA transcribed to cDNA and fluorescently labelled before microarray hybridization.

We normalized the data using RMA (Robust Multiple-array Average (4)), removed background fluorescence, and checked positive controls based on hybridization of Cy3-labeled spiked-in synthetic oligonucleotides.

Each condition was prepared in duplicates in two of the chambers, therefore the hybridization solution was prepared for a final volume of 140 µL with 62 µL per chamber. The mixture contained 70 µL of 2× MES buffer, 1.4 µL of herring-sperm DNA (10 mg/mL, Promega), 7 µL of acetylated BSA (10 mg/mL, Promega) and 4.67 µL of ‘QC 25mer’ (100 nM; Cy3-GAC CAG GGT GGT TCA TGA TGA TGA C) as an internal quality control. Nuclease-free water was added to the solution to reach a final volume of 140 µL. A 4-part self-adhesive hybridization chamber (Grace Bio-Labs Secure Seal RD475732-M) was placed on the array slide followed by addition of 62 µL of hybridization solution into each chamber and sealed with stickers. The array was incubated for 21 hours at 42°C under 300 rpm in the Thermo Shaker (IKA Matrix Orbital). Each individual chamber was washed with non-stringent wash buffer and afterwards immersed into a Petri dish with same buffer. The hybridization chamber was removed and transferred into a 50 mL falcon with same buffer content and washed for 2 minutes by vigorous shaking. Sequentially, another 50 mL falcon with stringent wash buffer for 1 minute and a final wash buffer for 10 seconds. The slide was dried using a microarray centrifuge and scanned at 2 µm resolution using a NimbleGen MS 200.

Quantitative PCR (qPCR) primers were designed using PCR Primer Design Tool from Eurofins Genomics. cDNA conversion was performed using same procedure as previously stated without labelling. A CFX Connect Real-Time PCR System (Bio-Rad) was used for the experiment. The qPCR master mix comprised 5 μL iTag Universal 2x SYBR Green Supermix (Bio-Rad), 1 μL forward and reverse primer (Table S4), 2 μL nuclease-free water, and 1 μL of diluted DNA, totaling 10 μL per reaction in a 96-well (low profile clear/clear) PCR plate, sealed with Microseal 'B' Sealing film (both Bio-Rad).

Each sample was run in duplicate, and each run included a standard and a negative control (nuclease-free water). Standard curves were generated using purified PCR products. The reactions followed this thermal profile: an initial hot-start activation at 95°C for 3 minutes, then 40 cycles of denaturation at 95°C for 10 seconds, annealing at 60°C for 30 seconds, and ending with a melting curve analysis. Absolute cell abundance was determined based on standard curves, with correction factors applied to account for multiple 16S rRNA gene copies in gut microbes (5).

### Lipidomics analysis

Briefly, pellets were resuspended in 225 µL of cold MeOH and vortexed for 20 seconds. Afterwards, samples were incubated in liquid nitrogen, thawed at RT and sonicated for 10 minutes at 4°C. This process was repeated 3 times to ensure cell lysis. Then, 750 µL of cold MTBE were added to the samples and incubated for 1 hour at 4°C with agitation. Then, 188 µL of water 0.1% ammonium acetate were added to allow phase separation and centrifuged at 10 000 g for 5 minutes. The upper part, corresponding to the lipid phase from the extraction, was collected and dried using speed vacuum (Christ PVC 2-25 CD plus, Christ CT 04-50SR, Edwards RV3 A65201903) for 2 hours and dissolved in 100 µL of 2-Prop/MeOH/CHCl3 (4:2:1) containing 7.5 mM ammonium acetate. The remaining phase was measured and added MeOH (4:1) to allow protein precipitation during 1 hour at -20°C, followed by a centrifugation at 13 000 g for 12 minutes at 4°C. Supernatants obtained were separated for the metabolite fraction, dried under speed vacuum overnight, and resuspended into 200 µL of acetonitrile/H_2_O (9:1), mixed, centrifugated 12 minutes at 13 000 g at 4°C to remove any cell debris and stored at -20°C. Continuing with protein fraction, pellets were dissolved in 200 µL of 1% SDS, 150 mM NaCl, 50 mM Tris (pH 7.8) and stored at –20°C. For the lipidomics analysis, the LC/MS method was used.


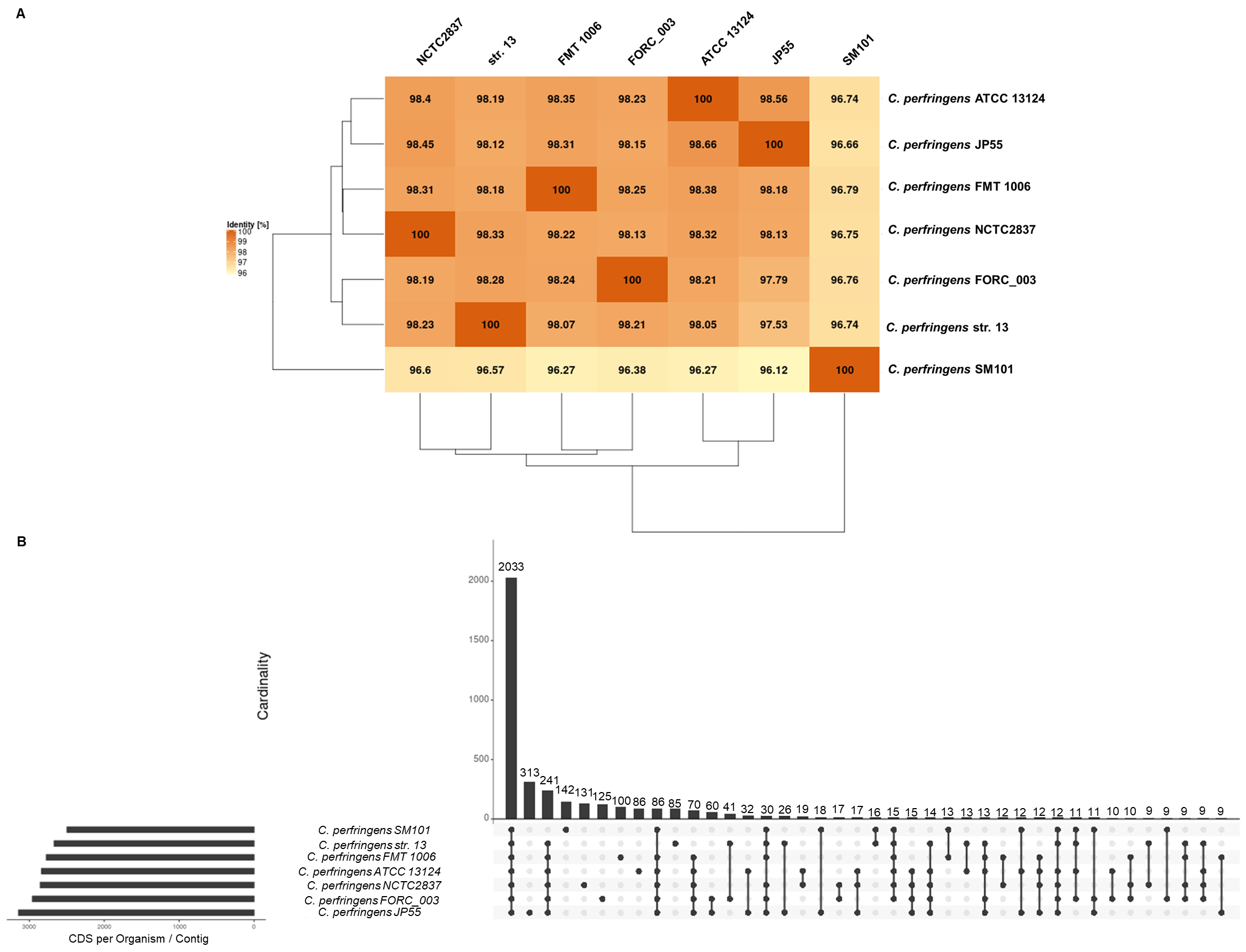


**Figure S1. Overall sequence identity and core genome.** (A) ANI heatmap with phylogenetic clustering based on BLASTn following Goris *et al.* method (6), darker color shows higher similarity. (B) UpSet plot of the pan-genome, core genome, unique CDS and shared CDS between each of the organisms.

**Table S1. Best hits of proteins encoded by pdu of C. perfringens FMT 1006.** BLASTp of the genes belonging to FMT 1006 against the database of NCBI using the database (non-redundant protein sequences). Shown are closest related proteins within Clostridium perfringens, the genus Clostridium, and genera other than Clostridium.

| Proteins from FMT 1006 blasted against the database of NCBI | | | | | | | |
| --- | --- | --- | --- | --- | --- | --- | --- |
| Protein | **Organism compared** | **GenBank identifier** | **E-value** | **CDS of FMT 1006** | **Score (bits)** | **Identities** | **Positives** |
| PcoR | *Clostridium perfringens* | WP_087325768.1 | 0 | T_01015 | 828 | 410/410 | 410/410 |
|  | *Clostridium baratii* | WP_039310678.1 | 2.00E-163 | T_01015 | 476 | 227/406 | 321/406 |
|  | *Eubacterium limosum* | WP_227208692.1 | 2.00E-108 | T_01015 | 336 | 180/413 | 262/413 |
| PduW | *Clostridium perfringens* | WP_003475280.1 | 0 | T_01799 | 812 | 398/398 | 398/398 |
|  | *Clostridium baratii* | WP_055206400.1 | 0 | T_01799 | 593 | 281/398 | 335/398 |
|  | *Thermobrachium celere* | WP_018661501.1 | 0 | T_01799 | 573 | 271/397 | 328/397 |
| DhaT | MULTISPECIES *Clostridium* | WP_003451991.1 | 0 | T_01024 | 791 | 385/385 | 385/385 |
|  | *Clostridium baratii* | MDU1855345.1 | 0 | T_01024 | 772 | 376/385 | 380/385 |
|  | *Rombutsia lituseburensis* | WP_258242312.1 | 0 | T_01024 | 684 | 326/384 | 353/384 |
| AdhE_2 | *Clostridium perfringens* | WP_003455710.1 | 0.00E+00 | T_00811 | 1003 | 492/492 | 492/492 |
|  | *Clostridium cadaveris* | WP_027640065.1 | 0 | T_00811 | 930 | 464/492 | 482/492 |
|  | *Romboutsia lituseburensis* | WP_270940972.1 | 0 | T_00811 | 744 | 361/492 | 418/492 |
| PduL | MULTISPECIES *Clostridium* | WP_003455694.1 | 2.00E-151 | T_00814 | 429 | 211/211 | 211/211 |
|  | *Clostridium tarantellae* | WP_152887939.1 | 1.00E-103 | T_00814 | 308 | 148/206 | 175/206 |
|  | *Clostridioides difficile* | ENY8712666.1 | 5.00E-102 | T_00814 | 304 | 142/208 | 172/208 |
| PduH | MULTISPECIES *Clostridium* | WP_003451934.1 | 8.00E-76 | T_01021 | 230 | 116/116 | 116/116 |
|  | *Paraclostridium bifermentans* | MDU3337673.1 | 5.00E-28 | T_01021 | 110 | 58/118 | 86/118 |
|  | *Romboutsia hominis* | WP_242871330.1 | 3.00E-27 | T_01021 | 108 | 57/113 | 80/113 |
| PduG | MULTISPECIES *Clostridium* | WP_003456360.1 | 0 | T_01020 | 1222 | 616/616 | 616/616 |
|  | *Clostridium chrysemydis* | WP_194191515.1 | 0 | T_01020 | 1031 | 526/608 | 574/608 |
|  | *Bacillus tuaregi* | WP_071392783.1 | 0 | T_01020 | 952 | 482/608 | 547/608 |
| PduE | MULTISPECIES *Clostridium* | WP_003451918.1 | 4.00E-95 | T_01019 | 281 | 141/141 | 141/141 |
|  | *Clostridium botulinum* | NFI51756.1 | 3.00E-70 | T_01019 | 218 | 109/140 | 119/140 |
|  | *Listeria monocytogenes* | EAE5653891.1 | 1.00E-62 | T_01019 | 199 | 99/140 | 115/140 |
| PduD | *Clostridium perfringens* | EHK2304691.1 | 2.00E-134 | T_01018 | 384 | 190/190 | 190/190 |
|  | *Clostridium baratii* | WP_224167811.1 | 1.00E-105 | T_01018 | 312 | 153/182 | 169/182 |
|  | *Listeria monocytogenes* | EIY5487079.1 | 4.00E-103 | T_01018 | 306 | 150/187 | 167/187 |
| PduC | MULTISPECIES *Clostridium* | WP_003451947.1 | 0 | T_01017 | 1137 | 553/554 | 554/554 |
|  | *Clostridium baratii* | WP_317411151.1 | 0 | T_01017 | 1014 | 479/554 | 524/554 |
|  | *Listeria monocytogenes* | EIY5487078.1 | 0 | T_01017 | 991 | 461/554 | 519/554 |

**Table S2. Comparison of end-products from PDU route between estimation and actual concentration in liquid phase.** YC-PD50: 1,2-propanediol. Estimated concentrations were calculated using Henry’s law constant from quantifications with PTR-TOF-MS and GCMS. Actual concentrations measured from liquid broth with HPLC-RI. Shown are average values from the biological duplicates.

|  | **Estimated concentration (mM)** | | **Actual concentration (mM)** | |
| --- | --- | --- | --- | --- |
| **Media** | **1-propanol** | **Propanal** | **1-propanol** | **Propanal** |
| **YC-PD50** | 14.1 | 10.1 | 7.5 | 1.9 |
| **YC-PD50** | 9.7 | 5.4 | 7.1 | 1.6 |

**Table S3. Differential expression of selected genes of *C. perfringens* dependent on culture conditions.** RNA isolated from *C. perfringens* FMT 1006 after 3-4 h culture in YC, YC-G50 and YC-PD50. RNA transcribed to cDNA labelled fluorescently and used in microarray hybridization. Log fold expression (LogFC) was calculated from fluorescence intensities at the tested conditions. * Indicates significancy (*p<0.05*) with Wald’s test. n.s: not significant.

| **genes of interest** | **locus_tag** | **YC vs. YC-PD50** | | | **YC-G50 vs. YC-PD50** | | |
| --- | --- | --- | --- | --- | --- | --- | --- |
|  |  | **logFC** | **adj.** **p. value** | **Change in expression level** | **logFC** | **adj.** **p. value** | **Change in expression level** |
| ***fabH*** | T_01163 | -0.8 to 1.0 | n.s | No change | -0.7 to 0.7 | n.s | No change |
| ***fabD*** | T_01164 | -0.4 to 0.9 | n.s | No change | -0.5 to 0.3 | n.s | No change |
| ***fabG_2*** | T_01165 | 0.1 to 0.4 | n.s | No change | -0.8 to 0.3 | n.s | No change |
| ***fabF*** | T_01166 | -0.3 to 0.0 | n.s | No change | -0.7 to 0.7 | n.s | No change |
| ***accB*** | T_01167 | -2.2 to 0.4 | 0.01* | Up in YC | -1.0 to 1.1 | n.s | No change |
| ***fabZ*** | T_01168 | -0.8 to 0.9 | n.s | No change | -0.4 to 0.3 | n.s | No change |
| ***cfiB*** | T_01169 | -1.0 to 0.3 | n.s | No change | -0.4 to 0.0 | n.s | No change |
| ***accD*** | T_01170 | -1.0 to -0.5 | n.s | No change | -2.0 to 0.8 | 0.00* | Up in Glucose |
| ***accA*** | T_01171 | -1.0 to 0.5 | n.s | No change | -0.7 to 0.6 | n.s | No change |

**Table S4. Primers designed for qPCR.** qPCR primers were designed using PCR Primer Design Tool from Eurofins Genomics. Standard curves were generated using purified PCR products. Efficiency was determined from standard curve.

| Primers designed | Forward (5’-3’) | Reverse (5’-3’) | Length product | Efficiency (%) |
| --- | --- | --- | --- | --- |
| *pduL_1* | TTATTTGGAGAAGGACATGAGC | TCTTTTAGCAACCATTAGCCC | 294 | 103.4 |
| *pduC_1* | CAAGAAGAACACCTGCTAACC | TCCTCTACCTACTTGTGAACC | 185 | 93.4 |
| *pduD_1* | ACAAGCTCCGCTTTTAACTC | ACTGGCTTTTTACCTGGTTC | 189 | 103.6 |
| *dhaT/pduQ_1* | CCACTTCCTCCAATAGTAGCAG | CAGCATGAGTTAAAGCGTCC | 214 | 88.5 |
| *gyrA_1* | GCTAAGGATAGAGCACTTCCAG | CCATCTCCATCTATACTTCCCC | 263 | 181.9 |
| *ftsZ_1* | GCCTTAACACTATCTCATGCC | AGCTCCAGTACCAGTACCAC | 195 | 99.4 |
| *recA_1* | AGCAGAGGCACAAAAATTAGG | TTGGAACTAAAGCGGCAAC | 215 | 147.0 |

### Literature

1. Das A, Santhosh S, Giridhar M, Behr J, Michel T, Schaudy E, et al. Dipodal Silanes Greatly Stabilize Glass Surface Functionalization for DNA Microarray Synthesis and High-Throughput Biological Assays. Anal Chem. 2023 Oct 17;95(41):15384–93.

2. Behr J, Michel T, Giridhar M, Santhosh S, Das A, Sabzalipoor H, et al. An open-source advanced maskless synthesizer for light-directed chemical synthesis of large nucleic acid libraries and microarrays [Internet]. ChemRxiv; 2024 [cited 2024 Aug 22]. Available from: https://chemrxiv.org/engage/chemrxiv/article-details/65ba15e39138d231611ab534

3. Lietard J, Leger A, Erlich Y, Sadowski N, Timp W, Somoza MM. Chemical and photochemical error rates in light-directed synthesis of complex DNA libraries. Nucleic Acids Res. 2021 Jul 9;49(12):6687–701.

4. Irizarry RA, Hobbs B, Collin F, Beazer‐Barclay YD, Antonellis KJ, Scherf U, et al. Exploration, normalization, and summaries of high density oligonucleotide array probe level data. Biostatistics. 2003 Apr 1;4(2):249–64.

5. Høgsgaard K, Vidal NP, Marietou A, Fiehn OG, Li Q, Bechtner J, et al. Fucose modifies short chain fatty acid and H2S formation through alterations of microbial cross-feeding activities. FEMS Microbiol Ecol. 2023 Oct 1;99(10):fiad107.

6. Goris J, Konstantinidis KT, Klappenbach JA, Coenye T, Vandamme P, Tiedje JM. DNA–DNA hybridization values and their relationship to whole-genome sequence similarities. Int J Syst Evol Microbiol. 2007;57(1):81–91.
